# Symmetry and spatial ability enhance change detection in visuospatial structures

**DOI:** 10.3758/s13421-022-01332-z

**Published:** 2022-06-15

**Authors:** Chuanxiuyue He, Zoe Rathbun, Daniel Buonauro, Hauke S. Meyerhoff, Steven L. Franconeri, Mike Stieff, Mary Hegarty

**Affiliations:** 1 Department of Psychological and Brain Sciences, University of California, Santa Barbara, CA 93106 USA; 2 University of Erfurt, Erfurt, Germany; 3 Leibniz-Institut für Wissenmedien, Tübingen, Germany; 4 Department of Psychology, Northwestern University, Evanston, IL USA; 5 University of Illinois, Chicago, IL USA

**Keywords:** Symmetry, Visuospatial working memory, Spatial ability, Change detection

## Abstract

Science, Technology, Engineering, and Mathematics (STEM) domains require people to recognize and transform complex visuospatial displays that appear to vastly exceed the limits of visuospatial working memory. Here, we consider possible domain-general mechanisms that may explain this advantage: capitalizing on symmetry, a structural regularity that can produce more efficient representations. Participants briefly viewed a structure made up of three-dimensional connected cubes of different colors, which was either asymmetrical or symmetrical. After a short delay, they were asked to detect a change (colors swapping positions) within a rotated second view. In change trials, the second display always had an asymmetrical structure. The presence of symmetry in the initial view improved change detection, and performance also declined with angular disparity of the encoding and test displays. People with higher spatial ability performed better on the change-detection task, but there was no evidence that they were better at leveraging symmetry than low-spatial individuals. The results suggest that leveraging symmetrical structures can help people of all ability levels exceed typical working memory limits by constructing more efficient representations and substituting resource-demanding mental rotation operations with alternative orientation-independent strategies.

**Supplementary Information:**

The online version contains supplementary material available at 10.3758/s13421-022-01332-z.

## Introduction

In classic visual working memory tasks, people can only maintain the colors of three to four separated geometric shapes (Luck and Vogel, 1997). Working memory capacity becomes drastically more limited when the task requires visuospatial transformations of these shapes such as rotations (Xu and Franconeri, 2015). However, science, technology, engineering, and mathematics (STEM) disciplines require people to think about more complicated visuospatial objects, such as mechanisms made up of many parts (Hegarty, 2004), molecules made up of many atoms (Stieff, 2007; Stieff et al., 2020), or complex geological structures (Hambrick et al., 2012), seemingly exceeding visuospatial working memory limits. One possibility is that this ability depends on recognizing domain-specific chunks (Chase and Simon, 1973; Goldstone et al., 2010; Morphew et al., 2015). However, we consider the alternative that it depends in part on the ability to capitalize on domain-general redundancies in stimuli to construct more efficient representations. We tested whether people might leverage symmetry in a visuospatial working memory task that involves detecting changes in a novel structure (i.e., a nonsense structure not dependent on any domain-specific knowledge) following a rotation. We call this task the *structure change-detection task* (Meyerhoff et al., 2021).

**Fig. 1:**
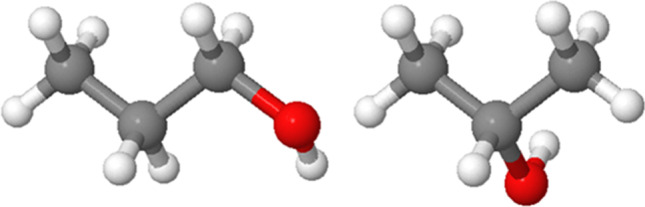
1-Propanol (left) and 2-propanol (right) both comprise three carbon atoms (gray), eight hydrogen atoms (white), and one oxygen atom (red). Because these molecules contain the same set of atoms, only connected in different ways, they are constitutional isomers; that is, they differ in the binding of visual and spatial properties

*Visuospatial* working memory requires binding visual properties to spatial locations (Treisman and Zhang, 2006; Wood, 2011). Visual-spatial bindings are central to the types of judgments that STEM students and professionals have to make about spatial structures. For example, in organic chemistry, students must learn to distinguish between molecules made up of the same atoms but with different connections between these atoms (see Fig. 1). True visuospatial working memory tasks contrast with those that primarily tap visual working memory, such as detecting a color or shape change in a change-detection task (e.g., Delvenne and Bruyer, 2006; Luck and Vogel, 1997; Vogel et al., 2001). They also contrast with tasks that primarily tap spatial working memory, such as the Corsi Blocks task, which involves storing and reproducing a sequence of block taps, defined by the relative location (a spatial property) of visually identical blocks.

Working memory capacity for visual feature-structure bindings is extremely limited (Alvarez and Thompson, 2009; Saiki, 2002; Scimeca and Franconeri, 2015; Xu and Franconeri, 2015). In a structure change-detection task in which people had to rotate a multipart object (a cross in which each of the four arms had a different color) and detected color swaps following rotation, Xu and Franconeri (2015) concluded that participants could only keep track of the color-structure binding of one part of the display. Even when the displays did not rotate, capacity for color-structure bindings was only about two, in contrast with spatially isolated simple visual features (e.g., color) for which the capacity is typically three to four.

Recent work in the visuospatial working memory literature has explored mental processes that help participants inflate their effective memory capacity. When items or patterns repeat (e.g., a display with multiple red items), viewers can use forms of compression to encode visual information more efficiently. Compression is a general-purpose strategy for storing more information in a limited capacity system, in which redundant information is stored only once, and the representation includes pointers to the positions of the redundant information (Brady et al., 2009; Meyerhoff et al., 2021). This possibility is supported by multiple studies in the visual working memory literature showing that stimulus regularities are associated with increased accuracy of participants in detecting changes to visual displays (Brady and Tenenbaum, 2013, Meyerhoff et al., 2021; Peterson and Berryhill, 2013; for a review, see Brady et al., 2009).

Here, we focus on the stimulus regularity of *symmetry*, which could also enable people to compress visual information for efficient encoding. Symmetry can be a salient property for the human visual system (Bertamini and Makin, 2014; Palmer, 1989; Royer, 1981) and is one of the Gestalt grouping principles (Brooks, 2015; Hartmann, 1935; Wertheimer, 1950). Symmetry is also an important property of structures in STEM disciplines, such as chemistry, structural engineering, and geology. For example, symmetry can affect molecular properties (see Fig. 1) or the structural integrity of a building, and geologists employ symmetry elements to imagine and interpret rock faults represented in block diagrams (Resnick and Shipley, 2013). Previous research has shown that performance on the Corsi Blocks task (spatial working memory) is improved when the configuration of blocks to be tapped is symmetrical (Rossi-Arnaud et al., 2006, 2012). However, to date no studies have examined the effects of symmetry on visuospatial working memory tasks involving the binding of visual features and spatial locations.

First, symmetry might support visuospatial working memory tasks by allowing for compression of mental representations. In this account, visual information is stored in a format that compresses information across symmetry. Compression over symmetry would enable people to encode more visuospatial information initially such that they have better performance in transforming (rotating) a symmetrical structure to detect changes.

Alternatively, it is possible that people might switch from mental rotation to an orientation-independent strategy when the two objects differ in symmetry. When asked to make judgments about objects following a rotation, researchers typically observe an increase in reaction time, and a decrease in accuracy, as a function of angular deviation of the objects to be compared. This is true in both simultaneous mental rotation paradigms (in which both objects are shown together, e.g., Just and Carpenter, 1985; Shepard and Metzler, 1971) and in sequential paradigms (where one object is displayed after the other, e.g., Bethell-Fox and Shepard, 1988; Cooper and Podgorny, 1976; Folk and Luce, 1987). This data pattern has been interpreted to indicate an analog process of attempting to rotate one object into congruence with the other (Cooper and Podgorny, 1976). However, tasks that involve making a judgment following a rotation do not necessarily require this analog “mental rotation” process. For example, in the Vandenberg and Kuse (1978) mental rotation test, people can use analytic strategies on some trials to eliminate answer choices (foils) that do not correspond to rotated views of the given object (Boone and Hegarty, 2017; Geiser et al., 2006; Hegarty, 2018). In these cases, the structures can be compared by using orientation-independent features (e.g., whether the two end arms of a structure are parallel or perpendicular). Moreover, these alternative strategies often involve encoding only part of the information available in the stimulus instead of the whole structure, which we refer to as *partial encoding*. When judgments are made on the basis of an orientation-independent feature, performance is not affected by angular disparity (Cohen and Kubovy, 1993; Takano, 1989).

A previous study in the domain of chemistry supports the possibility that symmetry enables people to switch from an analog mental rotation process to an analytic process. Stieff (2007) asked expert and novice chemists to decide whether representations of two molecules depict the same molecule or molecules that are mirror images of each other (structural isomers), essentially the same task as studied in Shepard and Metzler’s (1971) classic research on mental rotation. Chemistry novices solved this task by using mental rotation, such that reaction time increased with angular disparity. However, experts used an orientation-independent analytic strategy in which they first judged whether the molecules were symmetrical, knowing that if the molecule is symmetrical, it can always superimpose on its mirror image. In this instance, the experts were able to use a symmetry judgment to eliminate the need to perform a resource-intensive mental rotation, circumventing the limits of visuospatial working memory (cf. Hambrick et al., 2012). Here, we examine whether non-experts automatically detect and leverage symmetry of novel objects. That is, we examine whether non-experts switch from a mental rotation strategy to a symmetry change-detection strategy, noticing that the encoding stimulus is symmetrical and the test stimulus is not.

We also examine effects of spatial ability on the structure change-detection task. We predict that spatial ability will enhance performance on this task, based on the visuospatial nature of our task and research suggesting a strong relation between working memory capacity and cognitive abilities (Conway et al., 2003; Cowan et al., 2005; Hegarty and Waller, 2005; Lohman, 1988; Shah and Miyake, 1996; Unsworth et al., 2014). For example, Unsworth et al. (2014) found that measures of visual working memory capacity, independently of attentional control and secondary memory, accounted for the relation between working memory and general fluid intelligence. Here, we are concerned with the relation between working memory and spatial ability, rather than general intelligence, given that spatial ability uniquely predicts success in STEM independently of verbal and mathematical ability (Wai et al., 2009). We also explore whether high-spatial individuals are better than low-spatial individuals at capitalizing on symmetry to construct more efficient representations or to find alternative strategies, or whether the ability to capitalize on symmetry is independent of spatial ability.

## Experiment 1

**Fig. 2:**
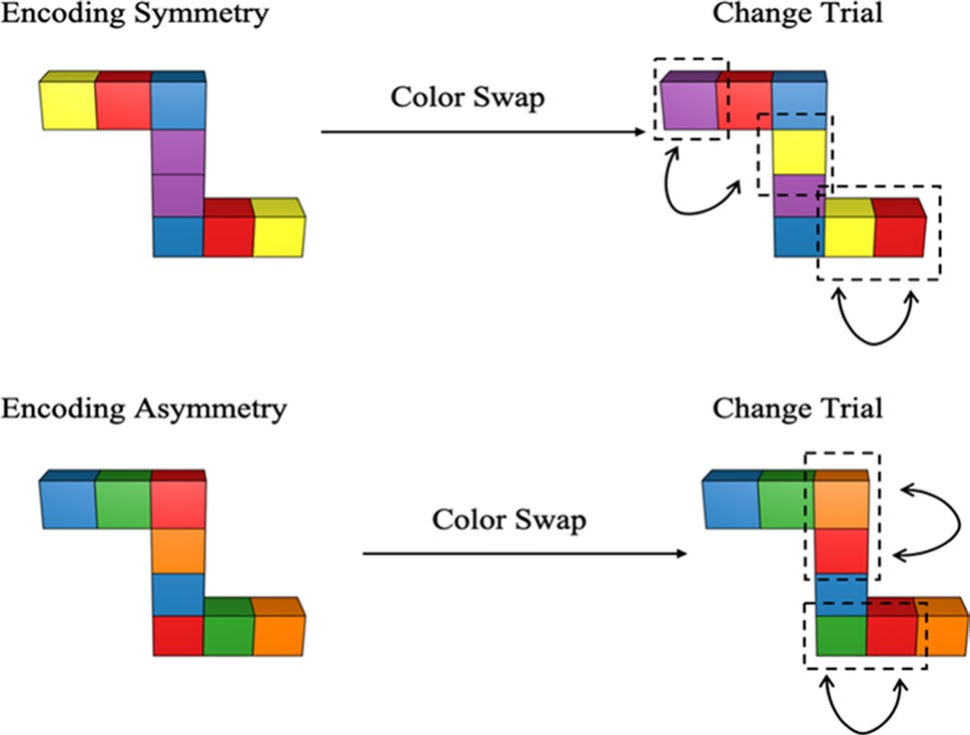
Examples of encoding stimuli (symmetrical or asymmetrical) and the corresponding test change stimuli for the structure detection task. All changes were color-swaps with two colors on each side swapped with each other. The resulting stimuli were always asymmetrical stimuli for the change trials

The task in Experiment 1 was structure change detection following a rotation. The structures were novel objects made up of eight connected cubes of different colors. While all objects had rotational symmetry with respect to shape, we varied the symmetry of the encoding stimuli such that some were symmetrical in colors while others were asymmetrical in colors (see Fig. 2). The test structures were always asymmetrical in change trials. To ensure that we were measuring visuospatial (rather than verbal) working memory, participants performed a concurrent verbal task.

We examine the mechanism underlying the advantage for symmetry change trials and contrast two hypotheses: (1) the compression hypothesis, that leveraging symmetry enables people to construct more efficient representations, facilitating change detection following a rotation, and (2) the analytic process hypothesis, that symmetry enables participants to substitute orientation-independent analytic processes for mental rotation. We tested the specific mechanisms by examining the angular disparity effect. Larger angular disparities involve increased difficulty in mental rotation (e.g., Boone and Hegarty, 2017; Shepard and Metzler, 1971) and object recognition (Gauthier et al., 2002; Lawson and Jolicoeur, 2003). We hypothesized that people would have poorer performance at detecting color-swaps with large angular disparity between the encoding and test view, at least when both the encoding and the test stimuli were asymmetrical. When the encoding stimuli were symmetrical, we might see a reduced effect of angular disparity (if symmetry supports the mental rotation process by allowing for compression) or no effect of angular disparity (if people base their response on a symmetry judgment rather than mental rotation, similar to the strategy adopted by experts in the study by Stieff, 2007).

When facing such complex objects, participants might use a partial coding strategy to encode the structure partially (e.g., the top half) and only detect changes within this part, ignoring the changes in the other parts. To mitigate the effects of this strategy, our stimuli were designed so that there was always color swap in both the top and the bottom halves of the stimuli, so that the chances of detecting a change were the same in symmetrical and asymmetrical stimuli. If the participant only encoded the top or bottom half, there should be no difference between change-detection performance for symmetrical and asymmetrical stimuli. Note that if a participant only encoded the top or bottom half of a stimulus, they might not detect that it was symmetrical.

Based on the visuospatial nature of our task, we hypothesized that people with more spatial ability would have superior performance in detecting color-swaps in general (capacity hypothesis). Spatial ability was measured by two commonly used tests of spatial visualization ability, the Cube Comparisons Test and the Paper Folding Test (Ekstrom et al., 1976; Hegarty and Waller, 2005; Lohman, 1988). We also explored the possibility that high-spatial participants would benefit more from symmetry.

### Method

#### Participants

Participants in Experiments 1 and 2 were recruited from an undergraduate subject pool and participated for course credit. All had normal or corrected-to-normal vision. Experiment 1 had 45 participants (27 females). Three participants (two females) were excluded from the analysis because they had lower than 80% accuracy on a verbal concurrent task (included to prevent verbal recoding of the stimuli, see below). Forty-two participants (25 females) were included in the final analysis.

A power analysis based on simulations (R package: simr, Green & MacLeod, 2016) indicated that the sample size in Experiment 1 enabled us to detect a small effect (*η*_*p*_^2^ = 0.01) for the main effects of symmetry, angle of rotation, and the rotation by symmetry interaction with power of (1- β) > .99 at α = .05 in a linear mixed model. We also have .9 power with an alpha level of .05 to detect a small main effect (*η*_*p*_^2^ = 0.01) for spatial ability (Brysbaert and Stevens, 2018) (see simulation in the shared data analysis code).

#### Materials

##### Stimuli and apparatus

Stimuli were presented on a 24-in. ASUS VG248 monitor with an AMD Radeon T R7450 graphics card, 1,920 × 1,080 resolution, 59-Hz refresh rate, and 8-bit depth. Stimuli were presented within a 20.6° region in the center of the computer monitor with a white background, and viewed at a distance of approximately 70 cm. In each trial, participants were shown a stimulus object composed of connected cubes (see Fig. 2) which subtended 7.6° × 6.3° of visual angle. Each stimulus was made up of eight cubes (two cubes of four different colors) with the four colors randomly selected from a set of six colors: red (RGB values: 228, 26, 28), orange (RGB values: 255, 127, 0), yellow (RGB values: 255, 255, 51), green (RGB values: 77, 175, 74), blue (RGB values: 55, 126, 184), and purple (RGB values: 152, 78, 163). The object was presented against a white (RGB values: 255, 255, 255) background. The encoding objects varied in whether the color binding of their constituent cubes was rotationally symmetrical or asymmetrical (see Fig. 2). Objects were created and rendered using Blender version 2.78. The addition of depth and shading meant that there was variation in the luminance values of the colors of the objects.

##### Structure change-detection task

A change-detection task procedure was employed in which participants were shown a set of two stimuli separated by a delay and judged whether the second (test) stimulus differed from the first (encoding) stimulus. The second stimulus was rotated by 10°, 60°, or 120° from the first. On half of the trials, the encoding and test stimuli were identical other than the 10°, 60°, or 120° rotation in the clockwise or counterclockwise direction (no-change trials). On the other half of trials, a swap in position of four of the eight cube units (two on each side) between the encoding and test stimulus in a rotated view indicated a “change trial.” For half of the trials, the first (encoding) stimulus was symmetrical, and for the other half, the first stimulus was asymmetrical. In change trials, the second stimulus was always asymmetrical. Thus, for half of the trials, the change included a change in symmetry (from symmetrical to asymmetrical), whereas for the others, the change was from one asymmetrical object to another asymmetrical object. There were 20 trials for each condition of the 2 (encoding symmetry) × 2 (change, no-change) × 3 (rotation) design for a total of 240 trials.

To prevent participants from encoding the structure verbally, a concurrent verbal task was employed. A letter string consisting of four random consonants was presented for 3 s before every trial. Participants were instructed to repeat these letters aloud through the entire trial. On 20% of trials, after making the judgment on the change-detection task, participants had to enter the repeated string. As feedback, the entered letters turned green (correct) or red (incorrect) for 500 ms. Participants whose accuracy was less than 80% on this were not included in the analyses.

##### Psychometric measures of spatial ability

The Paper Folding, Cube Comparisons, and Mental Rotation tests were administered. In the Paper Folding task (Ekstrom et al., 1976), participants were shown a depiction of a sheet of paper being folded in a series of steps. In the final step, a hole was shown to be punched in a certain location on the folded sheet of paper. The participants were asked to picture the configuration of holes that would result when the sheet of paper is unfolded, and to select which single depiction (of five answer choices) is correct. Participants completed two pages of ten problems each and were allowed 3 min to complete each page. The test score was the number of items solved correctly minus one-fourth of the number of items solved incorrectly.

In the Cube Comparisons task (Ekstrom et al., 1976), participants were shown depictions of two cubes with letters on each face of each cube. No letters were repeated on a given cube. The participants were to determine if the two cubes were the same or different (same cubes are different only by a rotation). Participants completed two pages of 21 items, and had 3 min to complete each page. The score on the test was the number of items solved correctly minus the number solved incorrectly.

We also included two versions of a spatial ability test, based on the Vandenberg and Kuse (1978) mental rotation test. These tests and results related to these tests are discussed in the Online Supplementary Materials (OSM). The Ishihara Compatible Pseudo Isochromatic Plate (PIPIC) Color Vision test (Waggoner, 2005) was used to test for color blindness.

#### Procedure

The local Institutional Review Board (IRB) reviewed and approved the study as adhering to ethical guidelines. After administration of the color blindness test, participants were given instructions for the structure change-detection task. The instructions explained that they would see two structures appear sequentially on the screen. After seeing the second structure, participants were to indicate if the two structures were the same or different. The instructions explicitly stated that the changes were color-swaps of four cubes making up the objects and stated that the second object would be rotated from the first, but did not mention symmetry or refer to the manipulation of angular disparity across trials. Participants were reminded that they should repeat the four letters throughout the trial and would be prompted to report the four letters on randomly selected trials. After reading the instructions, participants completed four practice trials. If they were not confident in their understanding, or performed poorly on these practice trials, they were asked to repeat the practice trials before proceeding.^1^

**Fig. 3:**
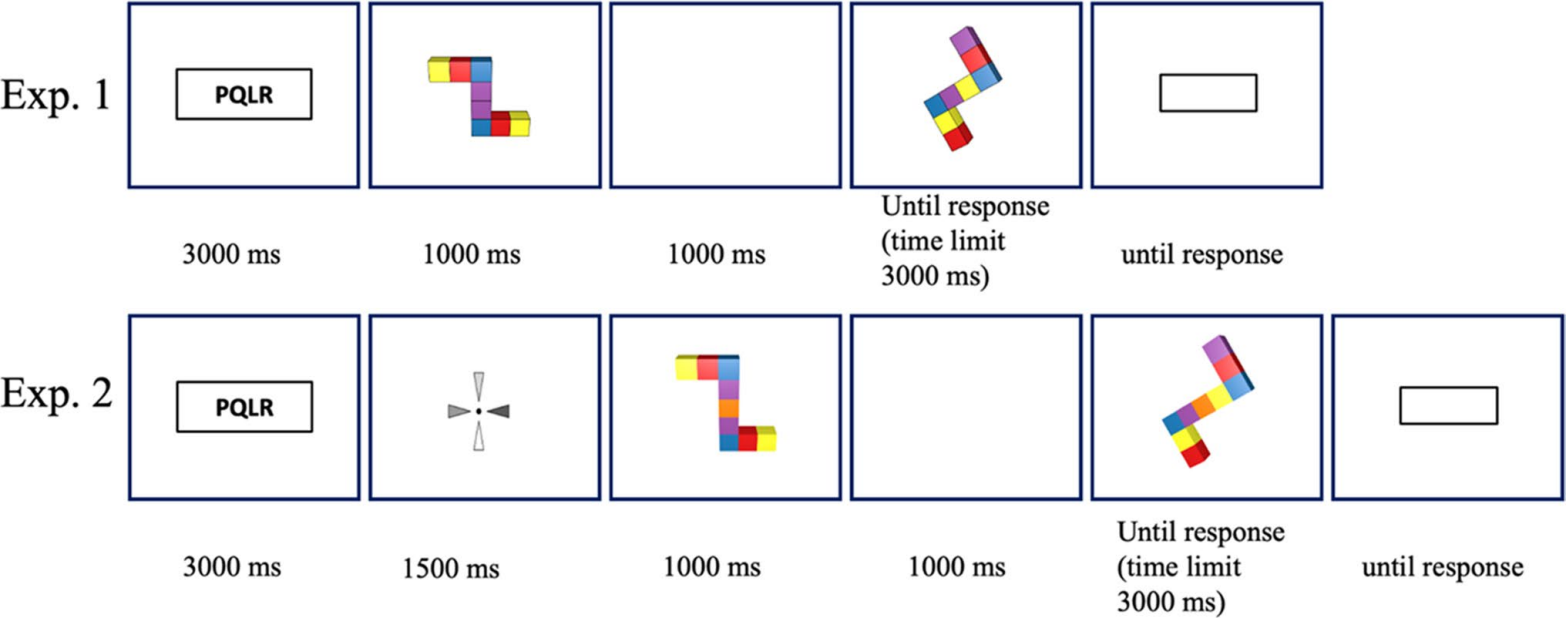
Procedure for the Structure Change-Detection Task. In Experiment 1, no rotation cue was presented; in Experiment 2, a rotation cue (indicating the direction and amount of rotation) to be imagined was added to each trial

Figure 3 depicts the structure change-detection task procedure. Participants needed to make the same or different judgments while doing the concurrent verbal task. Participants responded by pressing one of two keys (“1” for different, “9” for same) on a standard keyboard. After completing all trials of the structure change-detection task, participants were given the spatial ability measures.

### Results

#### Task performance

**Fig. 4:**
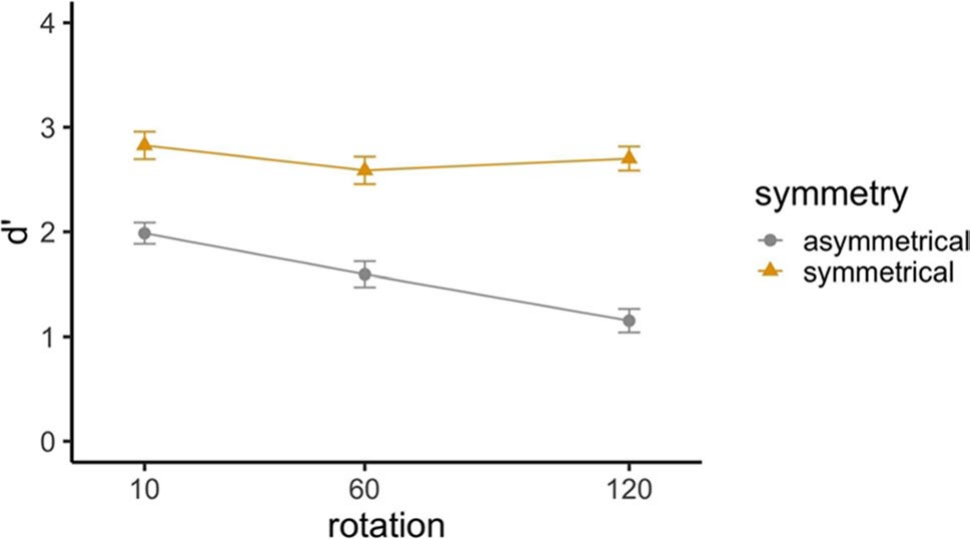
Sensitivity d’ by rotation angle and encoding symmetry for the Structure Change-Detection Task in Experiment 1

Accuracy as a function of encoding symmetry, rotation angle, and presence of a change is shown in Table 1.^2^ The table shows higher accuracy values for no-change than change trials, indicating a positive response bias. Therefore, additional analyses were conducted using d’ (i.e., sensitivity to detect changes, graphed in Fig. 4) as a measure of performance. Reaction time data (see full results in the OSM) showed similar patterns to the accuracy data.

**Table 1:** Means (standard errors in parentheses) for accuracy, response time (RT) and bias for Experiment 1

	Encoding symmetry	10°		60°		120°	
		Change	No change	Change	No change	Change	No change
Accuracy	Symmetrical	.86 (.02)	.94 (.01)	.83 (.02)	.92 (.01)	.89 (.01)	.91 (.01)
	Asymmetrical	.75 (.02)	.88 (.01)	.67 (.03)	.83 (.02)	.66 (.02)	.74 (.03)
RT (s)	Symmetrical	1.03 (.04)	1.01 (.03)	1.06 (.04)	1.08 (.04)	1.15 (.04)	1.11 (.04)
	Asymmetrical	1.17 (.04)	1.09 (.04)	1.21 (.04)	1.19 (.05)	1.24 (.04)	1.27 (.04)
Bias (*c*)	Symmetrical	1.95 (.22)		2.08 (.19)		1.56 (.17)	
	Asymmetrical	2.00 (.24)		2.08 (.26)		1.40 (.09)

Descriptive statistics for the psychometric measures are shown in Table 2. Paper Folding (PF) and Cube Comparison (CC) scores were significantly correlated (*r* = .51, *t*(40) = 3.73, *p* < .001, 95% CI [0.24, 0.70] ). Spatial ability was defined as the average of the z-transformations of these two spatial ability measures.

**Table 2:** Descriptive statistics for the psychometric measures in Experiments 1 and 2

	Mean	SD	Skewness	Kurtosis	Reliability^1^
Exp. 1					
Paper Folding	10.9	4.68	0.09	-1.22	0.75
Cube Comparison	16.79	9.24	-0.08	0.25	0.71
Exp. 2					
Paper Folding	11.47	4.38	-0.31	-0.6	0.71
Cube Comparison	17.37	8.06	0.15	-0.82	0.61
Verbal Reasoning	27.24	7.78	-0.83	0.11	0.84
Raven’s Progressive Matrices	10.12	2.82	-0.01	-0.99	-^2^

*Notes*.

^1^ Estimates of reliability are permutation-based split-half reliability estimation using r package splithalf. Data are repeatedly randomly split into two halves for 5,000 times. The final reliability is the average of the 5,000 split-half reliability estimates (Parsons et al., 2019)

^2^ The individual item scores for this administration of Ravens Progressive Matrices were lost due to technical issues but previous studies provide abundant evidence for high reliability and validity for this task (Raven et al., 1998)

#### Linear mixed model

A linear mixed model was used to test the effects of symmetry, angular disparity, spatial abilities, and their interactions on sensitivity to detect structure changes. We used R and lme4 (Bates et al., 2015) to perform the linear mixed model. The model had rotation angle, encoding symmetry, spatial ability, and three interactions (rotation and symmetry, symmetry and spatial ability, and rotation and spatial ability) as fixed factors. It also had random intercepts and slopes for participants. Rotation angles were centered at zero and scaled to have a standard deviation of one.^3^ P-values were obtained by likelihood ratio tests of the full model with the effect in question compared against the model without the effect in question. Table 3 shows a summary of the regression coefficients for the model.

**Table 3:** Coefficients table for the linear mixed model of Sensitivity for Experiment 1

Fixed effect	Estimate	Standard error	*χ*^2^	p-value	*η*_*p*_^2^
Rotation	-0.34	0.05	25.08	<.001***	0.43
Symmetry	1.13	0.08	192.50	<.001***	0.82
Spatial ability	0.26	0.09	9.54	.002**	0.20
Rotation × Symmetry	0.29	0.07	18.52	<.001***	0.31
Symmetry × SA	0.06	0.09	0.79	.38	0.02
Rotation × SÁ	-0.05	0.04	1.67	.20	0.04

List of fixed effects with coefficients, standard errors, *χ*^2 ^for likelihood ratio test, *p*-values, and effect size (*η*_*p*_^2^) from the linear mixed model. SA is short for Spatial Ability. Coefficients for interactions including Symmetry indicate the change from asymmetrical encoding to symmetrical encoding symmetry. Coefficients for interactions including Spatial Ability indicate the change from low Spatial Ability to high Spatial Ability

Participants were more able to detect changes in symmetrical trials than in asymmetrical trials, *χ*^2^(1) = 192.50, *p* < .001, with the symmetrical encoding trials increasing d’ about 1.13 (±0.08) compared to the asymmetrical encoding trials. Participants also had less sensitivity to changes with larger angular disparities (*χ*^2^(1) = 25.08, *p* < .001), with d’ decreasing by 0.34 (±0.05) for a 45° larger rotation. Further, a significant Rotation×Symmetry interaction revealed an effect of angular disparity for asymmetrical trials but not for symmetrical encoding trials. Specifically, the effect of angular disparity on d’ was -0.05 for symmetrical trials, which is not significantly different from zero.

As predicted, participants with higher spatial ability (measured by the Paper Folding and Cube Comparison tests) showed more sensitivity to changes (*χ*^2^(1) = 9.54, *p* = .002), with d’ increasing by 0.26 for people with 1 standard deviation higher spatial ability scores. However, we observed no significant interactions between spatial ability and Symmetry or Rotation. The Bayes factor (BF_10_ = 0.33) for the interaction term of symmetry by spatial ability indicates anecdotal evidence for the null hypothesis. BF_10_ = 0.33 for the interaction term of rotation by spatial ability, indicating anecdotal evidence for the null hypothesis.

### Discussion^4^

The results showed clear evidence for the benefit of symmetry in the structure change-detection task following a rotation. People were more able to detect changes on trials that included a change in symmetry (symmetrical encoding trials) compared to those that did not involve a change in symmetry (asymmetrical encoding trials). Experiment 1 also shows that people with better spatial ability were more sensitive to color-swaps in general. However, there was no evidence for differential effects of spatial ability for symmetrical and asymmetrical trials, that is, no evidence that spatial ability is related to the ability to take advantage of symmetry, indicating that symmetry – at least as operationalized in the present task – can benefit everyone, regardless of their spatial ability.

How does symmetry help people to exceed capacity limits? Sensitivity declined and response time increased with increases in angular disparity, consistent with studies of mental rotation (e.g., Cooper and Podgorny, 1976; Folk and Luce, 1987; Shepard and Metzler, 1971). However, this was true only for asymmetrical trials. Sensitivity d’ for symmetrical encoding trials was not significantly affected by angular disparity. This result suggests that people used an orientation-independent strategy, such as symmetry detection in the symmetrical encoding trials, consistent with the analytic process hypothesis. The use of an analytic process is consistent with claims by Shepard and Cooper (1982) and others (Takano, 1989) that the mental rotation process is only necessary when the task is to discriminate between an object and its mirror image. The data are not consistent with the compression hypothesis (which would result in a shallower slope, rather than the absence of an angular disparity effect).

Two features of the current experiment need to be taken into account in interpreting these results. First, there was no rotation cue in the change-detection task used in Experiment 1. In sequential mental rotation tasks, a rotation cue is typically shown before each trial to indicate the direction and amount of rotation to imagine, and this might be necessary to enable a mental rotation strategy. Moreover, it is possible that participants rotated the figures in the opposite direction to that intended, especially in the case of symmetrical-same trials, where a 120° clockwise rotation had the same sequence of colors as a 60° counterclockwise rotation. Second, in the case of symmetrical encoding structures, the two central cubes had the same color and were next to each other spatially (see Fig. 2), which is a very salient feature. Thus, on some trials, participants could detect a change from a symmetrical to an asymmetrical shape by just noting that the middle two squares change from being the same color to being different colors. These issues were addressed in Experiment 2.

## Experiment 2

Experiment 2 examines whether the benefits of symmetry and spatial ability can be replicated while addressing limitations of Experiment 1. First, Experiment 2 included a rotation cue. Second, to eliminate saliency of center repeated cubes, another cube was added to the figures in Experiment 2, so there were nine cubes for each stimulus and adjacent cubes were never the same color. Third, the results of Experiment 1 raised questions about whether participants used analytic processes rather than mental rotation, although the task involves detecting a change following a rotation. A post-task questionnaire was added to Experiment 2 to provide more information about their strategies. Finally, spatial ability tests showed positive correlations with detecting color-swaps, but it is important to examine whether this effect is due to spatial ability specifically, or whether it is a reflection of general intelligence, which is known to be related to visual working memory capacity (Unsworth et al., 2014). Therefore, a measure of general fluid intelligence, Raven’s Progressive Matrices (Raven et al., 1998), and a measure of verbal reasoning from the Differential Aptitudes test (Bennett et al., 1981), were added in Experiment 2.

### Method

#### Participants

Sixty-one (27 male, 34 female) students participated. Ten participants (four male, six female) were excluded from the analysis because they had lower than 80% accuracy on the verbal concurrent task and two participants (one male, one female) were excluded for not following instructions, leaving 49 (22 male, 27 female) participants in the final analysis.

Based on a power analysis, with 49 participants, we were able to detect a small effect (*η*_*p*_^2^ = 0.01) for the main effects of symmetry, angle of rotation, and the rotation by symmetry interaction with power of (1- β) > .99 at α = .05 in a linear mixed model. We also have .88 power with an alpha level of .05 to detect a small main effect (*η*_*p*_^2^ = 0.01) for spatial ability (see simulation in the shared data analysis code).^5^

#### Materials

##### Stimuli and apparatus

**Fig. 5:**
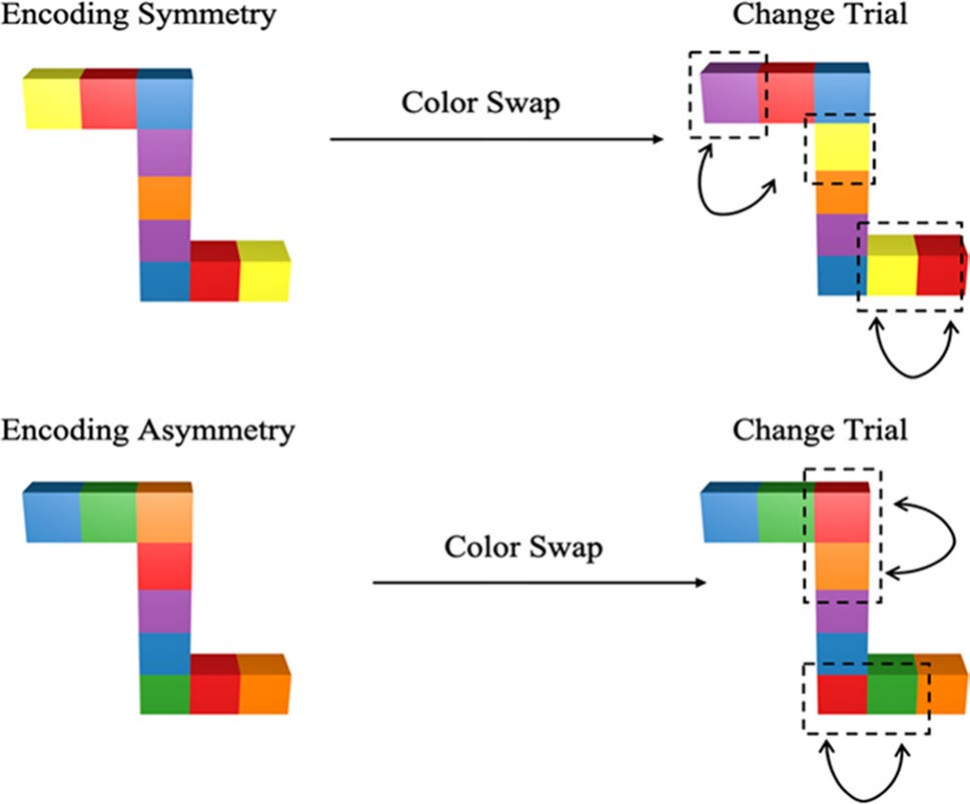
Examples of encoding stimuli (symmetrical or asymmetrical) and the corresponding test with-change stimuli for the change-detection task. All changes were color-swaps with two colors on each side swapped with each other. The resulting stimuli were always asymmetrical stimuli

The stimuli were similar to those in Experiment 1, except that they were made up of nine cubes, two cubes of four different colors and one central cube of a fifth color (see Fig. 5). The colors were chosen from the same set of colors as in Experiment 1 and subtended 7.6° × 6.3° of visual angle. The apparatus was identical to Experiment 1

##### Structure change-detection task

The structure change-detection task was the same as in Experiment 1 except that the rotation to be imagined on each trial was indicated by a rotating wheel, which appeared after the test stimulus and rotated by the same angle as the figure (10°, 60°, or 120°; see Fig. 3).

As in Experiment 1, the study had a 2 (encoding symmetry) × 2 (change, no change) × 3 (rotation: 10°, 60°, 120°) design with 20 iterations of each stimulus type for a total of 240 trials. On half of the trials, the encoding and test stimuli were identical other than the rotation, and in the other half they were different (change trials). As in Experiment 1, on change trials, two cubes were swapped on each half of the stimulus as defined by the point of rotational symmetry. Half of the encoding objects were symmetrical and half were asymmetrical, while all of the test stimuli were asymmetrical.

##### Ability measures

As in Experiment 1, the Paper Folding and Cube Comparisons tasks were administered as measures of spatial ability and the Ishihara Compatible Pseudo Isochromatic Plate (PIPIC) Color Vision test (Waggoner, 2005) was used to test for color blindness.

A short form of the Raven’s Advanced Progressive Matrices, Set II (RAPM) consisting of the 18 odd numbered items of the original test (Raven et al., 1998) was administered as a test of General Fluid Intelligence (g). Each problem consists of a 3 × 3 grid of images with the final bottom-most right corner image missing. These grids or matrices contain a pattern or “rule” that the participant must discover to select the missing image out of the six answer choices. Participants were allowed 10 min to complete 18 items. The Verbal Reasoning subtest of the Differential Aptitudes Test (Bennett et al., 1981) was administered as a test of verbal reasoning and consists of verbal analogies (e.g., dog is to bark as cat is to meow). Participants were asked to choose the pair that best completes the sentence. The task consists of three sample questions and 40 problems, and participants were allowed 15 min to complete the test.

##### Post-task questionnaire

The post-task questionnaire consisted of three open-ended questions about strategies, which started with non-direct, open-ended questions and ended with a more direct question about symmetry, as follows:

1. “Please describe strategies, if any, you used during today’s task”.
2. “Did you notice any patterns in the structures that you were presented with today?”
3. “Did you notice the symmetry during the task? If you did notice the symmetry, did you think it influenced how you approached the structure task?”

#### Procedure

After performing the color blindness test, participants were given instructions for the structure change-detection task, which were the same as in Experiment 1, except that participants were explicitly informed that the second object would be shown at a different angle, with the angular disparity indicated by the rotation of a wheel that appeared before the first object. As in Experiment 1, the instructions made no reference to symmetry. The sequence of events in each structure change-detection trial is shown in Fig. 3. The only difference from Experiment 1 was the presentation of the rotating “windmill,” indicating the amount of rotation to be imagined on that trial. The amount of rotation was always equivalent to this amount.

After completing the structure change-detection task, participants were administered the Paper Folding and Cube Comparisons spatial ability measures, Raven's Advanced Progressive Matrices (RAPM), DAT Verbal Reasoning test and the strategy questionnaire, in that order.

### Results

#### Task performance

**Fig. 6:**
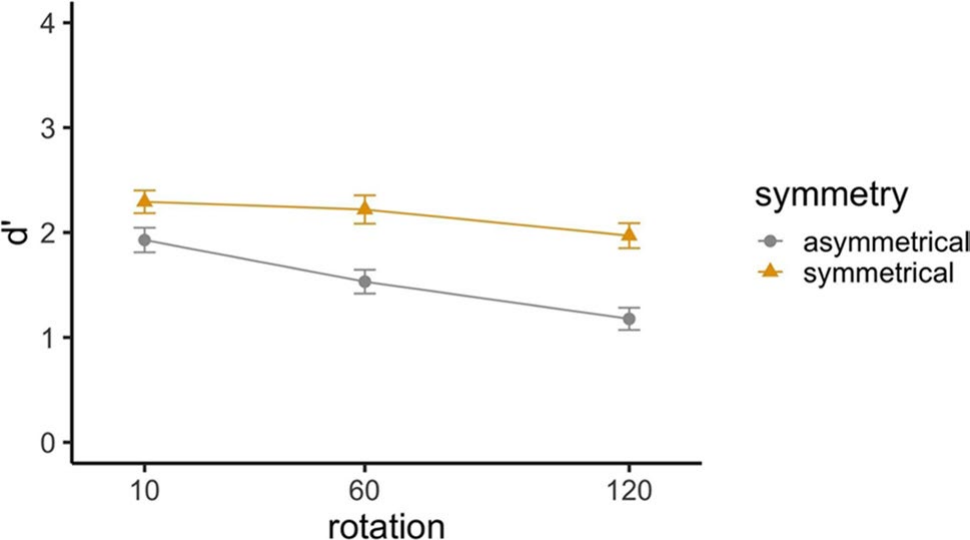
Sensitivity d’ for the structure change-detection task in Experiment 2. Line graphs of d’ by rotation angle and encoding symmetry

Accuracy as a function of encoding symmetry, rotation angle, and presence of a change are shown in Table 4. As in Experiment 1, there was a positive response bias, so additional analyses were conducted using d’ (i.e., sensitivity to stimulus changes; graphed in Fig. 6) as a measure of performance.

**Table 4:** Means (standard errors in parentheses) for accuracy, bias, and response time for Experiment 2

		10°		60°		120°	
	Encoding symmetry	Change	No change	Change	No change	Change	No change
Accuracy	Symmetrical	.81 (.02)	.89 (.01)	.79 (.02)	.88 (.02)	.76 (.02)	.86 (.02)
	Asymmetrical	.71 (.03)	.88 (.02)	.68 (.03)	.82 (.02)	.66 (.02)	.75 (.02)
RT (s)	Symmetrical	1.16 (.04)	1.15 (.04)	1.23 (.04)	1.21 (.04)	1.25 (.04)	1.25 (.04)
	Asymmetrical	1.19 (.04)	1.17 (.04)	1.25 (.04)	1.23 (.04)	1.29 (.04)	1.34 (.04)
Bias (*c*)	Symmetrical	1.74 (.16)		1.55 (.15)		1.66 (.14)	
	Asymmetrical	2.32 (.23)		1.61 (.14)		1.51 (.18)

Descriptive statistics for the psychometric measures are presented in Table 2.^6^ As in Experiment 1, the two spatial abilities measures (Cube Comparison and Paper Folding) were significantly correlated with each other (*r* = .39, *t*(47) = 2.93, *p* = .005, 95% CI [0.12, 0.61]) and the measure of spatial ability was the average of the z scores for these two ability measures. This measure of spatial ability was significantly correlated (*r* = .52, *t*(47) = 4.17, *p* < .001, 95% CI [0.28, 0.70]) with Raven's Progressive Matrices but not significantly correlated (*r* = .21, *t*(47) = 1.48, *p* = .15, 95% CI [-0.07, 0.46]) with Verbal Reasoning.

#### Linear mixed model

A linear mixed model was fitted to the data to examine effects of encoding symmetry, rotation, and cognitive abilities on sensitivity in the structure change-detection task. The model-fitting procedure was the same as Experiment 1 except for adding verbal reasoning ability (VR) and general intelligence (RAPM) as covariates. Table 5 shows a summary of the regression coefficients for the model.

**Table 5:** Coefficients table for the linear mixed model of Sensitivity for Experiment 2

Fixed effect	Estimate	Standard error	*χ*^2^	p-value	*η*_*p*_^2^
Rotation	-0.31	0.04	50.53	<.001***	0.51
Symmetry	0.62	0.08	56.74	<.001***	0.55
Spatial ability	0.44	0.11	13.54	<.001***	0.16
Verbal reasoning	-0.09	0.08	1.11	.29	0.02
RAPM	0.02	0.09	0.06	.81	0.001
Symmetry × Rotation	0.17	0.06	9.66	.002**	0.07
SA × Rotation	0.01	0.04	0.05	.83	<0.001
SA × Symmetry	-0.18	0.09	3.87	.05*	0.07

List of fixed effects with coefficients, standard errors, *χ*^2 ^for likelihood ratio test, p-values, and effect size (*η*_*p*_^2^) from the linear mixed model. SA = Spatial Ability. Coefficients for interactions including Symmetry indicate the change from asymmetrical encoding to symmetrical encoding symmetry. Coefficients for interactions including Spatial Ability indicate the change from low Spatial Ability to high Spatial Ability

As in Experiment 1, people were more sensitive to changes in symmetrical trials than asymmetrical trials (*χ*^2^(1) = 56.74, *p* < .001), with the symmetrical encoding trials increasing d’ about 0.62 (±0.06) compared to the asymmetrical encoding trials. Participants again had less sensitivity to changes with larger angular disparities (*χ*^2^(1) = 50.53, *p* < .001), with d’ decreasing by 0.31 (±0.04) for a 45° larger rotation. We observed a significant Rotation ×Symmetry interaction (*χ*^2^(1) = 9.66, *p* = .002), revealing a smaller but significant effect of angular error for symmetrical trials compared to asymmetrical trials with d’ decreasing to 0.14 (±0.04) for a 45° larger rotation for symmetrical trials.

As in Experiment 1, people with higher spatial ability showed greater sensitivity to changes (*χ*^2^(1) = 13.54, *p* < .001), with d’ increasing by 0.44 for people with 1 standard deviation higher spatial ability scores. Again, we observed no significant interaction between spatial ability and rotation, and the Bayes factor (BF_10_) for this interaction term is 0.2, indicating moderate evidence that the interaction is not significant. However, we observed a marginally significant interaction of spatial ability and symmetry, which indicates that symmetry affected the performance of low-spatial individuals more than that of high-spatial individuals. The Bayes factor (BF_10_) for this interaction of symmetry by spatial ability is 3.04, indicating moderate evidence for this interaction. Neither verbal reasoning nor RAPM had significant effects. These results provide no evidence that verbal reasoning ability and general intelligence account for additional variation in this task over and above spatial ability.

#### Strategy reports

We first examined the open-ended strategy reports. Initial inspection of these reports indicated that in addition to mental rotation and symmetry detection, participants reported a “partial encoding” strategy of focusing on a subset of the information in the stimuli, so we also coded reports of this strategy. Two authors independently coded the responses, yielding consistency of 89% across the three questions, and a third resolved discrepancies. Table 6 includes the final code list and frequency counts.

**Table 6:** List of reported strategies in the open-ended strategy reports for Experiment 2

Strategy	Count (percentage)	Representative Self-Reports
Symmetry	17 (34.7%)	“I looked at the ends and middle of the figure to see if there was any symmetry”; “It was easier to see if both the top and bottom matched”
Partial coding	45 (91.8%)	“I also tried to focus on the brighter colors in the sequence; look at “the middle of the structure” or “top three cubes”
Mental rotation	12 (24.5%)	“I would also attempt to see whether the three colors I focused on did in fact move the direction in which the arrow pointed” “I would tilt my head a little to help recognize and compare it from the first image”.

The majority of participants reported partial encoding strategies, but only a few spontaneously mentioned mental rotation as a strategy. About one-third of participants spontaneously mentioned using symmetry in the first open-ended strategy question. In the follow-up questions that prompted students about patterns in the stimuli and symmetry specifically, most (36 students, 73.5%) reported that symmetry helped them perform the structure-detection task. However, most of these (26 students, 53%) commented that they noticed symmetry of only parts of the stimulus (e.g., “I only paid attention to the symmetry of the first three color blocks”). Only a minority (ten participants, 20.5%) referred to symmetry of the whole structure (e.g., “the structure was symmetrical based on color”). There was no evidence of differences in performance between those who did or did not mention symmetry or mental rotation, and we did not have the power to detect whether the partial coding strategy affected performance, considering that all but five of the participants reported a version of this strategy.

### Discussion

Experiment 2 replicated the benefits of symmetry and spatial ability found in Experiment 1. Again, there was no evidence that high spatial individuals benefited more from symmetry. If anything, the trend toward the interaction points in the opposite direction, namely that low spatials may benefit more. Experiment 2 also showed that people with better spatial ability were more sensitive to changes (d’), even after controlling for measures of fluid intelligence and verbal reasoning ability, suggesting that the advantages are specific to spatial ability.

When the encoding stimulus was symmetrical, performance was less influenced by angular disparity. However, in contrast to Experiment 1, there was a small but still significant effect of angular disparity for symmetrical stimuli. The presence of the rotation cue or reduced salience of symmetry due to the central cube may have increased the use of mental rotation as a strategy in this experiment. The difference in slopes for symmetrical and asymmetrical encoding stimuli suggests that even if participants used mental rotation as a strategy, they rotated a compressed or partially encoded stimulus when the stimulus was symmetrical (compression hypothesis). Alternatively, they might have used an orientation-independent (analytic) process such as detecting symmetry on some but not all symmetrical trials.

Strategy self-reports supported the conclusion that symmetry was used by most people, although they also suggested that the majority of participants only paid attention to parts of the structures (using partial encoding strategies). Note that if the partial encoding strategy is the only strategy used by participants, their performance on symmetrical and asymmetrical trials should not differ, because swaps occurred in both halves of the structure. Thus, by only looking at half of the structures, they would have the same chance of detecting differences, suggesting that other processes, besides partial encoding, also influenced performance.

## General discussion

The present experiments examine the effect of symmetry on a visuospatial working memory task that involves detecting changes to color-structure bindings. Our results demonstrate an advantage of symmetry in both experiments, such that participants had higher sensitivity in detecting color-swaps when these changes altered the symmetry of the object, even in a rotated view. Participants with higher spatial ability, as measured by Paper Folding and Cube Comparisons, showed better performance in general, and this relationship held even after controlling for general intelligence and verbal reasoning ability. However, there was no evidence that people with high spatial ability benefited more from symmetry changes. In general, symmetry detection benefitted participants of all spatial ability levels, and if anything, benefited those with low spatial abilities more.

We outlined two potential mechanisms underlying the benefits of symmetry. First, it is possible that people compress visuospatial information based on symmetry such that they encode all visuospatial bindings in an efficient way. According to this compression hypothesis, more efficient representations require less storage capacity, and thus facilitate the mental rotation process by allowing more working memory resources for processing. Second, people might detect changes by noticing a symmetry change, which is an analytic (orientation-independent) process. In Experiment 1, there was no angular disparity effect for the symmetry change trials, which is more parsimoniously explained by the analytic process. However, in Experiment 2, after adding a rotation cue and a middle cube to reduce the salience of symmetry, performance declined with angular disparity for both symmetrical and asymmetrical stimuli, although this effect was stronger for asymmetrical encoding trials. This result is consistent with the compression account, but also with the possibility that people used the analytic process inconsistently. It is supported by the result that response times were more variable in Experiment 2 (see OSM), which is consistent with the use of different strategies. It is possible that due to the additional middle cube, participants failed to detect the symmetry on all relevant trials, that the presence of the rotation cue encouraged a mental rotation process, or both.

Strategy self-reports indicated an interesting tactic of selectively attending to a subset of the information in the stimulus (i.e., partial encoding). For example, some people reported focusing on certain colors (e.g., yellow) to detect changes in the spatial locations of these colors. Others reported only focusing on part of the structure (e.g., the top row or midsection) to detect color changes at these locations. Note that a partial encoding strategy is not only useful for symmetrical trials. For asymmetrical trials, if participants only focus on half of the structure, their performance should be as good as that for symmetrical trials, because there is a color swap in both halves. However, their performance was actually better for symmetrical trials, indicating that partial encoding is not the only strategy they used, although it is the one most often reported. It is possible that symmetrical trials encourage people to use the partial encoding strategy, and this boosts performance in symmetrical trials relative to asymmetrical trials.

More generally, while our task involved structure change detection following a rotation, and performance declined with angular disparity (at least for asymmetrical encoding trials), the cognitive processes underlying this effect may not be mental rotation. First, although performance decreased as the angular disparity between the encoding and test stimuli increased, this decrease in performance was less evident when the encoding stimulus was symmetrical and was not significantly different from zero in Experiment 1. Second, very few of the participants reported deliberately rotating the encoding stimulus to compare it with the test stimuli in a rotated view. In classic mental rotation tasks, the judgment is to detect whether the two stimuli are the same or mirror images (i.e., a “handedness” judgment). In contrast, the foils in this present research involved color-swaps. Previous research has indicated that people are less likely to perform mental rotation when the foils involve changes other than mirror images (Boone and Hegarty, 2017; Cheung et al., 2009). Our research is consistent with this, and with the view that tasks that involve detecting a change following a rotation do not necessarily measure the analog imagery process known as “mental rotation” (Gauthier et al., 2002; Hayward et al., 2006). With the feasibility of alternative strategies, people exploit these strategies instead of mental rotation or other cognitively demanding holistic strategies.

Spatial ability, measured by paper folding and cube comparisons tests, gave a general advantage in the structure change-detection task, which was observed in both sensitivity to a change and in response times (see OSM). This result is consistent with findings that (1) people with high spatial ability tend to have better performance on tasks that depend on spatial working memory (Shah and Miyake, 1996; Miyake et al., 2001), and (2) working memory capacity predicts performance in higher-order cognition tasks (e.g., Conway et al., 2003; Unsworth et al., 2014). However, there was no interaction of spatial ability with either symmetry or rotation angle in either experiment. Specifically, we found no support for the hypothesis that the ability to leverage symmetry to store or manipulate spatial representations is a component of spatial ability, at least for the type of simple rotational symmetry tested here, leaving this explanation as less likely to explain why people with high spatial ability have good performance in STEM domains. In fact, Experiment 2 indicated preliminary evidence that, if anything, those with low spatial ability benefited more from symmetry. It is possible that low spatial participants depended relatively more on symmetry detection because they were less able to use a mental rotation strategy. However, we stress that these results are preliminary, as the present experiments did not have sufficient power to detect interactions of spatial ability with the experimental variables and these effects should be studied further in future studies with more power to detect these interactions.

This paper studied one type of symmetry (i.e., rotational symmetry in terms of structure-color bindings). Different types of symmetry (e.g., mirror symmetry) have different levels of detectability and both the axis of symmetry and frequency with which a given symmetry axis is shown also affect how well symmetry is detected (Julesz, 1971; Wagemans, 1997). Future studies should also examine the effects of symmetry of the structure itself (i.e., geometric symmetry) on mental rotation and change detection. Interestingly, recent studies highlighted effects of geometric symmetry in larger-scale spatial cognition such that symmetry (in the alignment of the buildings with the structure of an environment) influenced how well people could integrate information across different locations in a large-scale environment, and spatial working memory moderated ability to achieve this integration (He et al., 2019, 2021). The present study shows that rotational symmetry of color bindings boosts people’s performance in detecting structure changes in a rotated view, which highlights a new direction of research to investigate the effects of other types of symmetry on visuospatial working memory tasks, including extensions to other scales of space.

Overall, this research study is the first to show that non-experts in STEM can take advantage of symmetry to tackle a visuospatial working memory task (i.e., structure change detection across rotation) with complex but novel stimuli. Spatial ability, which benefits STEM students in other contexts, also shows general positive influence on performance in the structure change-detection task. However, leveraging symmetry appears to involve a set of domain-general mechanisms including information compression, selective attention, and analytic thinking processes that do not appear to depend on spatial ability. These results offer promising applications to STEM education in that they identify domain-general strategies that can be capitalized on in future educational programs to boost students’ success in STEM fields, regardless of their spatial abilities.

## Supplementary Information

ESM 1 (DOCX 300 kb)

## Data Availability

The datasets generated during and/or analyzed during the current study will be available on Github: https://github.com/CarolHeChuanxiuyue/LinearMixedModel_VWMStudy.git None of the experiments was preregistered.

## Footnotes

1 Only one participant had to repeat the practice trials in Experiment 1 and three participants had to repeat them in Experiment 2 (two of them repeated the practice trials once and one repeated them twice).

2 See OSM for histograms of the distributions for each measure.

3 Centering and scaling all predictors in the linear regression was implemented to reduce multicollinearity given that the model contains multiple interaction terms. The original values of the rotation, 10°, 60°, and 120° were transformed to -1.18, 0.07, and 1.25. Moreover, it makes the coefficients of the predictors more comparable to each other. The predictors have different scales but the spatial ability, Raven’s Progressive Matrices and Verbal Reasoning are all z-scored. Centering and scaling the rotation degree can also be treated as standardization of degrees of rotation so that all coefficients can be treated as standard coefficients and the values of the coefficients are not influenced by the scales of different predictors.

4 The linear mixed models on the response time are in general consistent with the conclusions based on the sensitivity in Experiments 1 and 2. Thus, the main text only reports the models for sensitivity. See OSM for the detailed analysis and discussions on the response time.

5 Both simulations in Experiment 1 and Experiment 2 assumed a minimum effect size of η_p_^2^ = 0.01 for all predictors and normal distributions for the continuous predictors. The design matrix of the categorical predictors (symmetry and rotation) is the same as the experimental design.

6 Only Cube Comparisons showed a sex difference, *t*(44.8) = -2.02, *p* = .05, d = .58, where men had a slightly better performance. With sample size of 49, we do not have sufficient power to detect sex differences.

## Code Availability

The R scripts for analyzing the datasets will be available on Github: https://github.com/CarolHeChuanxiuyue/LinearMixedModel_VWMStudy.git

The software application used for running the task is available from the corresponding author on reasonable request.
