# Supplementary material for: Symmetry and spatial ability enhance change detection in visuospatial structures
Source: Mem Cognit. 2022 Jun 15;50(6):1186–200. doi: 10.3758/s13421-022-01332-z (PMC9365739; doi:10.3758/s13421-022-01332-z)
Supplement: Supplementary file 1 — (DOCX 300 kb) [file 13421_2022_1332_MOESM1_ESM.docx]

# Supplemental Materials

### Mental Rotation Task

Two versions of the Vandenberg & Kuse (1978) Mental Rotation Test (adapted by Boone & Hegarty, 2017) were administered in Experiment 1. Each item consisted of a row of five block figures. The left-most figure was the standard figure. Two of the four figures to the right were different from the goal figure by a rotation. The other two figures could not be rotated into congruence with the goal figure. The participant’s task was to mark the two figures that could be rotated to match the goal figure. In one version (MRT_M for mirror), all foils were mirror images of the standard figures. In the other version (MRT_S for structure), all foils were structurally different. Each version had 2 sections of 10 items (a total of 40 items). Participants are allowed 3 minutes for each section of the test. The score was the number of items on which both correct figures were marked.

The correlations of the two versions of the MRT with other spatial measures and d’ were similar (see Table S1), and the correlation between the two MRT tasks (MRT_M and MRT_S) was 0.71 (*t*(40) = 6.40, *p* <.001, 95%*CI* for r [.52,.83]). Thus, we averaged the z-scores of the two scores to indicate mental rotation ability (MRT) for the following analysis. There were no sex differences in mental rotation but with a sample of 17 men versus 25 women, we had limited power to detect sex differences in this experiment.

As shown in Table S1, the MRT score was not significantly correlated with d’ on the structure change detection task, although it is significantly correlated with scores in Paper Folding and Cube Comparisons. Moreover, including z-transformed MRT scores in the spatial ability composite scores did not affect the significance of any variables in the linear mixed model of sensitivity for Experiment 1.These results supports the conclusion in the main text that mental rotation might not be the dominant strategy used in the structure change detection task in Experiment 1.

*Table S1*

*﻿Correlation table for the tasks in Experiment 1 including Mental Rotation.*

|  | d’ | Paper Folding | Cube Comparisons | MRT_M |
| --- | --- | --- | --- | --- |
| Paper Folding | .33* |  |  |  |
| Cube Comparisons | .47** | .51*** |  |  |
| MRT_M | .21 | .46** | .51*** |  |
| MRT_S | .20 | .50*** | .45** | .71*** |

Note: * stands for *p* < .05, **: *p* < .01, ***: *p* < .001

### Response Times

Response time analyses were based on correct trials completed within the 3 second time limit per trial. In Experiment 1, participants timed out on 1.0% of trials and responded incorrectly on 16.7% of trials. In Experiment 2, participants timed out on 0.6% of trials and responded incorrectly on 28.8% of trials. All timed-out trials and trials with incorrect responses were removed from the response time analysis.


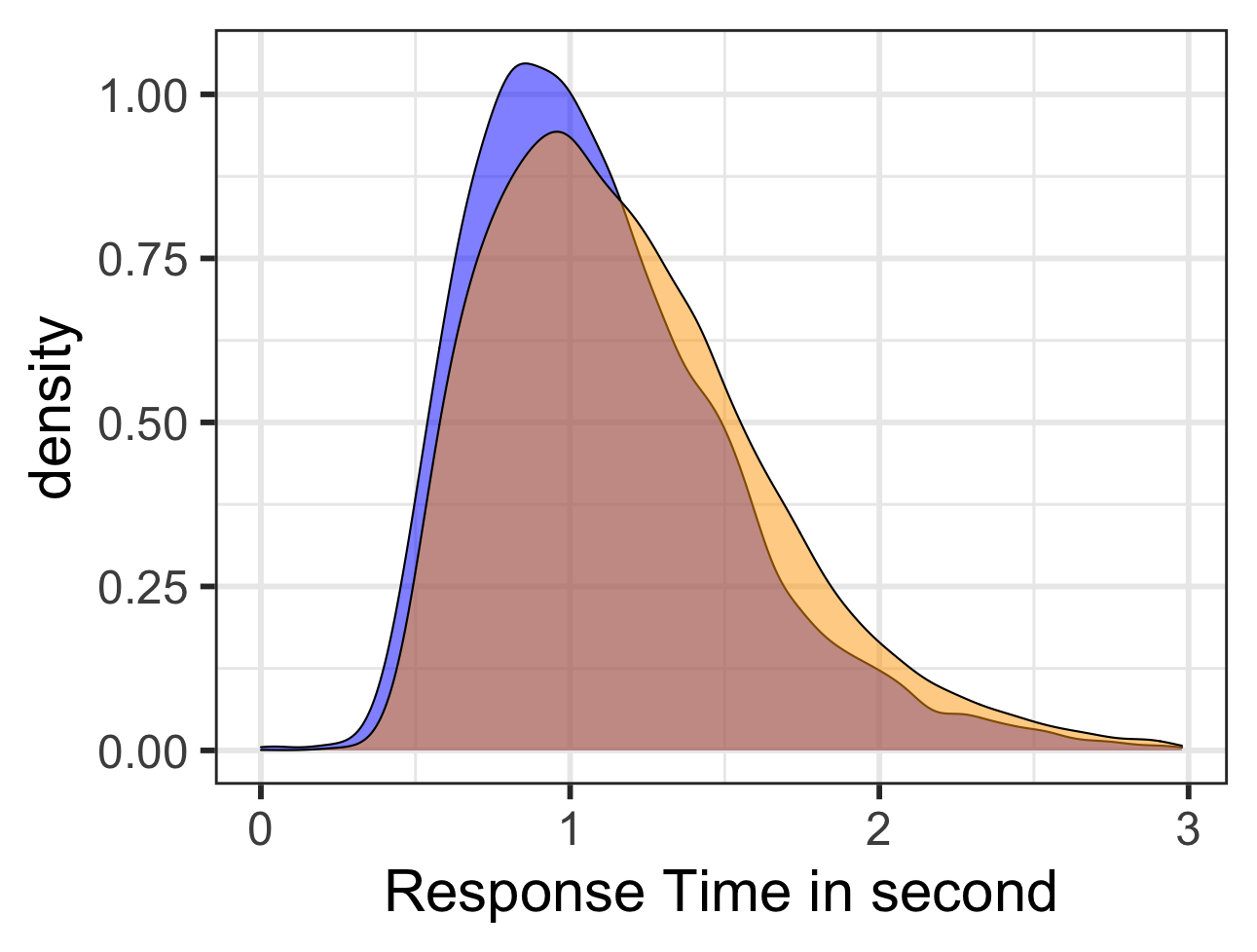

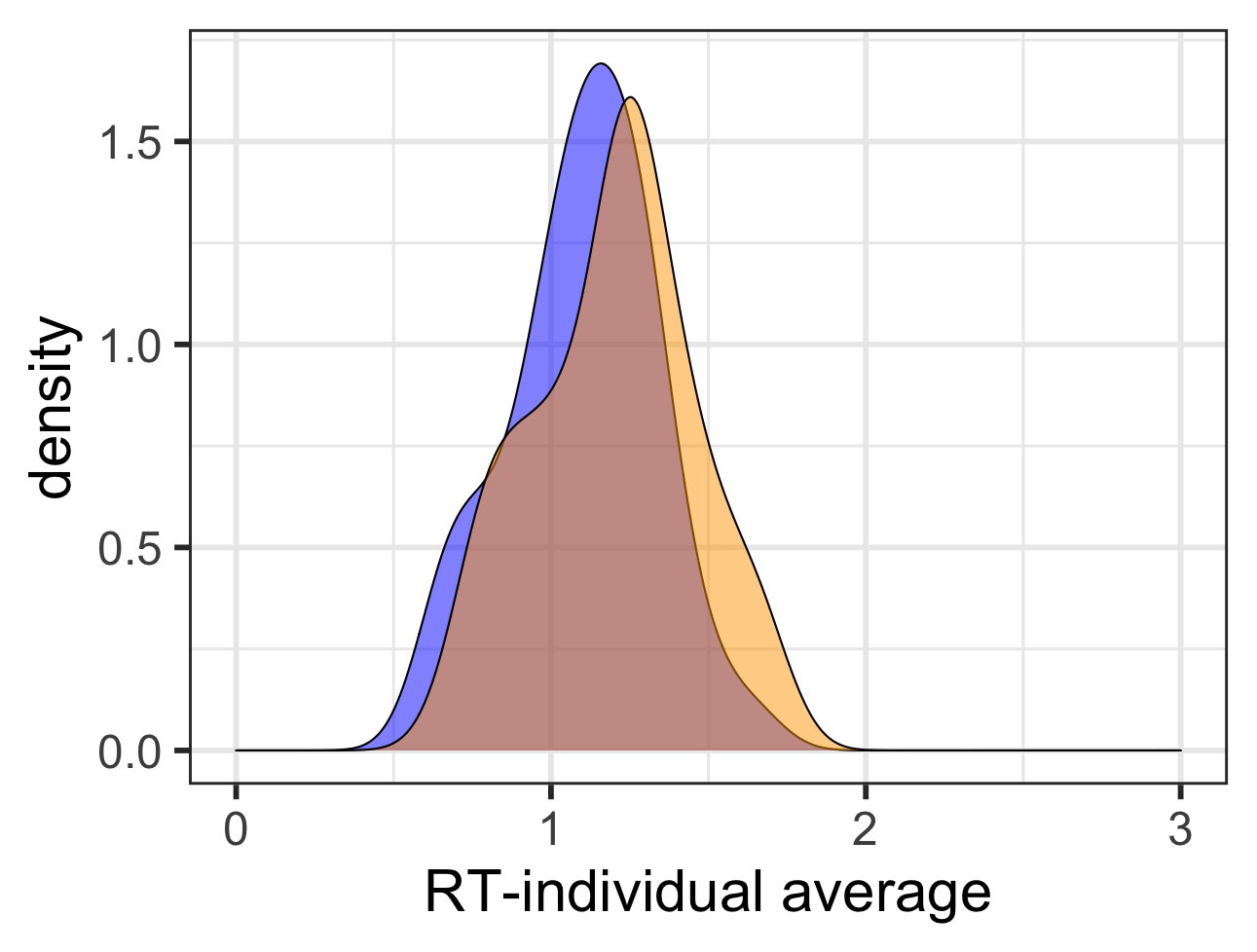

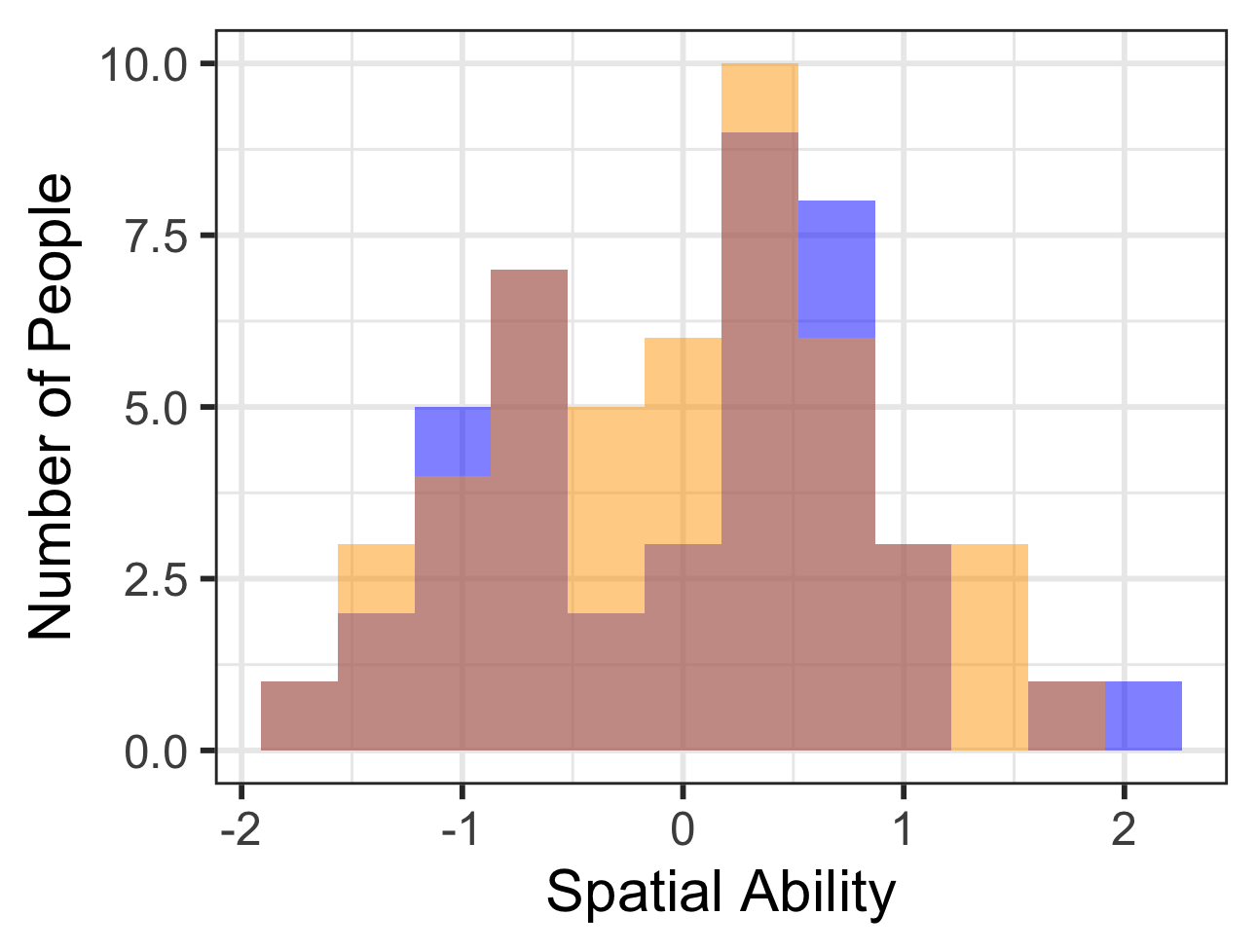

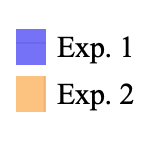


(a) (b) (c)

Figure S1. (a) The distributions of the response time across all trials in two experiments (purple for Exp. 1, orange for Exp. 2); (b) The distributions of the average RT for each participant in two experiments; (c) The distributions of the Spatial Ability for each participant in two experiments.

As shown in Figure S1 (a. trial level; b. individual level), response times were in general slower in Experiment 2 (Mean = 1.19s) than in Experiment 1 (Mean = 1.10s), likely reflecting the figure was more complex (made up of 9 cubes rather than 8) and that symmetry was more difficult to detect. In addition, the rotation cue might have encouraged people to rely more on mental rotation and less on the orientation-independent analytic strategy (i.e., symmetry detection). This interpretation is consistent with the conclusions based on sensitivity reported in the manuscript. Response times had a larger variance in Experiment 2 (*SD* = 0.45s) than in Experiment 1 (*SD* = 0.42s), suggesting that participants were more likely to shift their strategies within the tasks in Exp. 2. This is consistent with conclusions based on the sensitivity analysis and self-reported strategy analysis. Specifically, given the reduced salience of symmetry and the rotation cue, people might use the analytic process, mental rotation, and partial encoding inconsistently.

Correlations between individual differences measures in the two experiments are shown in Table S2.

*Table S2*

*﻿Correlation table for individual measures in Experiment 1 & Experiment 2*

| **Experiment 1** |  |  |  |
| --- | --- | --- | --- |
|  | **Spatial Ability** | **d’** | **Response Time - mean** |
| **d’** | 0.46** | - |  |
| **Response Time - mean** | 0.07 | -0.03 | - |
| **Experiment 2** |  |  |  |
|  | **Spatial Ability** | **d’** | **Response Time - mean** |
| **d’** | 0.48*** | - |  |
| **Response Time - mean** | -0.43** | -0.38** | - |
| **Verbal Reasoning** | 0.21 | -0.01 | 0.005 |
| **Raven’s** | 0.52*** | 0.23 | -0.22 |

Note: * stands for *p* < .05, **: *p* < .01, ***: *p* < .001

The average response time was significantly correlated with participants’ spatial ability in Experiment 2 (*r* = -.43, *t*(47) = -3.24, *p* =.002, 95%*CI* for r [-.63,-.17]) but not in Experiment 1 (*r* = .07, *t*(40) = 0.49, *p* =.63, 95%*CI* for *r* [-.23, .37]). It is consistent with conclusions in the main text that participants used more analytic strategies (symmetry detection) in Experiment 1 than in Experiment 2 in which they relied more on mental rotation, which depends more on spatial ability.

***Linear Mixed Models for Response Times***

Linear mixed models tested the effects of symmetry, angular disparity, change type, their interactions and spatial ability on response time in the two experiments. In Experiment 1, (see Table S3), there was a significant effect of symmetry such that participants responded faster (by 110 msec) for symmetrical compared to asymmetrical encoding trials. Moreover, response time increased with angular disparity, and a significant interaction of symmetry and rotation, indicating an effect of angular disparity, which was reduced for symmetrical stimuli. In general participants took more time in conditions where they showed less sensitivity to a change, indicating that there was no speed-accuracy tradeoff and conclusions based on response times are consistent with those based on the sensitivity analysis.

*Table S3*

*﻿Coefficients table for the linear mixed model of Response Time for Experiment 1*

| Fixed Effect | Estimate | Standard Error | $\chi^{2}$ | *p*-value | ${\eta_{p}}^{2}$ |
| --- | --- | --- | --- | --- | --- |
| Rotation | 0.08 | 0.01 | 52.40 | <.001*** | 0.57 |
| Symmetry | -0.11 | 0.02 | 58.58 | <.001*** | 0.68 |
| Change | 0.01 | 0.02 | 0.62 | .43 | .004 |
| Spatial ability | 0.001 | 0.04 | 0.001 | .98 | <.001 |
| Symmetry$\times$  Rotation | -0.04 | 0.01 | 7.01 | .010** | 0.14 |
| Change$\times$Rotation | -0.05 | 0.01 | 21.62 | <.001*** | 0.15 |
| Symmetry$\times$Change | -0.04 | 0.02 | 7.78 | .005** | 0.08 |
| Rotation$\times$Symmetry$\times$Change | 0.03 | 0.02 | 2.42 | .11 | 0.04 |

*Note:* List of fixed effects with coefficients, standard errors, $\chi^{2}$for likelihood ratio test, *p*-values, and effect size (${\eta_{p}}^{2}$) from the linear mixed model. SA is short for Spatial Ability. Coefficients for interactions including Symmetry indicate the change from asymmetrical encoding to symmetrical encoding symmetry. Coefficients for interactions including Spatial Ability indicate the change from low Spatial Ability to high Spatial Ability. Change indicates the change from no-change trials to change trials.

In Experiment 2 (see Table S4) people responded faster for the symmetrical trials and trials with less angular disparity, mirroring the sensitivity analysis so that, again, there is no speed-accuracy tradeoff. Moreover, although changes were smaller than that in Experiment 1, there is still a significant interaction of symmetry and rotation, which is consistent with the use of a symmetry detection process in Experiment 2, although less frequently than in Experiment 1.

*Table S4*

*﻿Coefficients table for the linear mixed model of Response Time for Experiment 2*

| Fixed Effect | Estimate | Standard Error | $\chi^{2}$ | *p*-value | ${\eta_{p}}^{2}$ |
| --- | --- | --- | --- | --- | --- |
| Rotation | 0.07 | 0.01 | 90.02 | <.001*** | 0.61 |
| Symmetry | -0.05 | 0.02 | 6.86 | .01** | 0.16 |
| Change | 0.01 | 0.02 | 1.60 | .21 | 0.01 |
| Spatial ability | -0.13 | 0.04 | 11.98 | <.001 | 0.20 |
| Symmetry$\times$  Rotation | -0.03 | 0.01 | 4.65 | .03* | 0.07 |
| Change$\times$Rotation | -0.03 | 0.01 | 5.96 | .01* | 0.09 |
| Symmetry$\times$Change | 0.01 | 0.02 | 0.20 | .65 | 0.004 |
| Rotation$\times$Symmetry$\times$Change | 0.02 | 0.02 | 1.86 | .17 | 0.03 |

*Note:* List of fixed effects with coefficients, standard errors, $\chi^{2}$for likelihood ratio test, *p*-values, and effect size (${\eta_{p}}^{2}$) from the linear mixed model. SA is short for Spatial Ability. Coefficients for interactions including Symmetry indicate the change from asymmetrical encoding to symmetrical encoding symmetry. Coefficients for interactions including Spatial Ability indicate the change from low Spatial Ability to high Spatial Ability. Change indicates the change from no-change trials to change trials.

Comparing the model for Experiment 1 (Table S3) with the model for Experiment 2 (Table S4), the key differences are 1) spatial ability had a significant effect in Experiment 2 but not in Experiment 1; 2) symmetry by change effect was significant in Experiment 1 but not in Experiment 2. These results are consistent with the conclusions in the main text that the structure change detection in Experiment 2 was more demanding of spatial ability due to the reduced saliency of symmetry and the added rotation cue encouraging mental rotation.
